# Autoimmune GFAP astrocytopathy with eosinophils on cerebrospinal fluid cytology and isolated spinal cord lesions on MRI: a case report

**DOI:** 10.3389/fimmu.2026.1865920

**Published:** 2026-06-16

**Authors:** Dongdong Zhang, Yahan Song, Yongjie Bai, Junqiang Yan, Ruile Shen

**Affiliations:** Department of Neurology, The First Affiliated Hospital, and College of Clinical Medicine of Henan University of Science and Technology, Luoyang, Henan, China

**Keywords:** autoimmune GFAP astrocytopathy, case report, cerebrospinal fluid cytology, cerebrospinal fluid eosinophilia, isolated spinal cord lesions, myelitis

## Abstract

**Background:**

Autoimmune glial fibrillary acidic protein (GFAP) astrocytopathy is an autoimmune inflammatory disorder of the central nervous system associated with GFAP-IgG. It most commonly presents as meningoencephalitis, myelitis, or meningoencephalomyelitis. Although MRI abnormalities in the brain and spinal cord are common, isolated spinal cord lesions without corresponding brain MRI abnormalities are uncommon and may pose a diagnostic challenge. Eosinophils identified on cerebrospinal fluid cytology have rarely been reported in this disorder.

**Case presentation:**

A 31-year-old woman presented with fever, headache, urinary retention, and meningeal irritation signs. Despite these findings, brain magnetic resonance imaging (MRI) was unremarkable, whereas spinal MRI revealed discontinuous patchy long-segment intramedullary lesions in the thoracic cord. Cerebrospinal fluid (CSF) analysis showed elevated opening pressure, pleocytosis, increased protein, and 10% eosinophils on cytological examination. Infectious studies, including CSF culture and metagenomic next-generation sequencing, were negative. Serum and CSF antibodies against aquaporin-4, myelin oligodendrocyte glycoprotein, and myelin basic protein were negative, whereas CSF GFAP-IgG was positive at a titer of 1:32, while serum GFAP-IgG was negative. Following high-dose intravenous methylprednisolone and an oral prednisone taper, the patient showed marked clinical, CSF, and radiological improvement, with complete resolution of thoracic cord lesions on follow-up MRI.

**Conclusion:**

Isolated spinal cord lesions on MRI may represent an important clue to autoimmune GFAP astrocytopathy and should prompt consideration of this diagnosis even in the absence of brain MRI abnormalities. The presence of eosinophils on cerebrospinal fluid cytology may further suggest a distinct inflammatory profile and offer insight into the pathophysiology of the disease.

## Introduction

Autoimmune glial fibrillary acidic protein (GFAP) astrocytopathy is an autoimmune-mediated inflammatory disease of the central nervous system defined by GFAP-IgG positivity, predominantly in cerebrospinal fluid (CSF) (1, 2). Since its recognition as a distinct clinical entity, an expanding spectrum of neurological manifestations has been described, including meningoencephalitis, myelitis, and meningoencephalomyelitis (3, 4). Despite increasing awareness, diagnosis remains challenging because of the heterogeneity of clinical presentation and neuroimaging findings.

MRI abnormalities involving the brain and spinal cord have been reported in many patients with autoimmune GFAP astrocytopathy. Nevertheless, some cases show atypical clinicoradiological dissociation, such as prominent meningeal symptoms with unremarkable brain MRI or isolated spinal cord involvement. These nonclassical patterns may delay recognition and lead to diagnostic confusion with infectious or other autoimmune inflammatory disorders. CSF findings in autoimmune GFAP astrocytopathy usually include lymphocytic pleocytosis and elevated protein concentration. Eosinophils detected on CSF cytology are distinctly uncommon (5, 6) and may further complicate the diagnostic process by suggesting alternative etiologies, such as parasitic infection or eosinophilic meningoencephalitis. At the same time, this rare finding may reflect a unique inflammatory milieu and provide clues to the pathophysiology of the disease.

In this report, we describe a patient with autoimmune GFAP astrocytopathy characterized by eosinophils on CSF cytology, isolated thoracic spinal cord MRI lesions, meningeal irritation signs, and normal brain MRI findings. This case emphasizes the need to consider autoimmune GFAP astrocytopathy in patients with isolated inflammatory spinal cord lesions and highlights the potential relevance of CSF eosinophils in understanding disease mechanisms.

## Case presentation

A 31-year-old woman was admitted with a 5-day history of fever and headache and a 1-day history of urinary retention. Five days before admission, she developed a persistent fever without an obvious trigger, with a peak temperature of 38.6 °C, accompanied by headache. She had no cough, sputum production, or other respiratory symptoms. One day before admission, she developed difficulty urinating and came to our hospital. She had no previous neurological disease, autoimmune disease, allergic disease, malignancy, or known immunodeficiency. Her family history was negative for neurological, autoimmune, allergic, or malignant diseases. She had no history of parasitic infection, residence in or travel to parasite-endemic areas, exposure to contaminated water, intake of raw or undercooked high-risk foods, or relevant animal contact. She denied recent drug exposure, new medication use, or vaccination before symptom onset. On admission, her temperature was 38.3 °C, pulse rate 83 beats/min, blood pressure 114/69 mmHg, and respiratory rate was 16 breaths/min. Neurological examination on admission showed that the patient was alert and fully oriented, with no disturbance of consciousness or cognitive impairment. Nuchal rigidity and Kernig’s sign were present, whereas Brudzinski’s sign was negative. Cranial nerve examination was unremarkable. There was no clinical evidence of optic nerve involvement, including visual loss, eye pain, visual field defect, or abnormal pupillary light reflex. Motor strength was Medical Research Council grade 5/5 in all four limbs, and muscle tone was normal. Deep tendon reflexes were normal and symmetric. Pyramidal signs, including bilateral Babinski signs, were absent. Sensory examination revealed no definite sensory level and no objective impairment of superficial or deep sensation. Coordination testing was normal, and gait was not impaired. The predominant spinal cord-related manifestation was acute urinary retention, consistent with involvement of spinal autonomic pathways regulating bladder function.

Routine blood tests and coagulation studies were within normal limits. Peripheral blood eosinophils were within the normal range. ANCA was negative, and the erythrocyte sedimentation rate was not elevated. Serum cytokine testing, including IL-2, IL-4, IL-6, IL-8, and IL-10, was negative. Gynecological ultrasonography showed no evidence of ovarian teratoma or other gynecological mass. Nucleic acid testing of throat swab specimens was negative for influenza A and B viruses, parainfluenza virus, human metapneumovirus, Mycoplasma pneumoniae, Chlamydia pneumoniae, respiratory syncytial virus, adenovirus, and rhinovirus. Serologic tests for syphilis and human immunodeficiency virus were negative. Tests for Mycobacterium tuberculosis ESAT-6 and CFP10 antigens were also negative. Brain MRI, including post-gadolinium T1-weighted sequences, showed no abnormal findings. In particular, there was no linear radial perivascular enhancement, leptomeningeal enhancement, ependymal enhancement, or abnormal parenchymal enhancement. Thoracic spinal MRI showed discontinuous, patchy, longitudinally extensive intramedullary lesions in the upper-to-middle thoracic cord, approximately from T2 to T6. The lesions were hypointense on T1-weighted images and hyperintense on fat-suppressed T2-weighted images. Axial T2-weighted images showed that the lesions were predominantly central within the cord, mainly involving the central/periependymal region, without an eccentric or peripheral white-matter tract predominance. There was no obvious cord swelling, mass effect, or compressive lesion (**Figures 1A–D**). Lumbar puncture showed an opening pressure of 400 mmH_2_O. CSF analysis revealed a leukocyte count of 260/mm^3^, a protein level of 1017 mg/L, a glucose level of 2.31 mmol/L, and a chloride level of 119 mmol/L. Cytological examination with May–Grünwald–Giemsa staining showed 85% lymphocytes, 5% monocytes, and 10% eosinophils (**Figure 2**). CSF culture was negative, and metagenomic next-generation sequencing of the CSF did not identify any pathogen. GFAP-IgG was tested using a cell-based assay (CBA) performed by Zhengzhou KingMed Center for Clinical Laboratory, Zhengzhou, Henan, China. No tissue-based assay was performed. According to the laboratory report, the assay was based on a cell-transfection indirect immunofluorescence method, in which mammalian cells transfected with the GFAP antigen expression construct and co-expressing green fluorescent protein were used as the antigen substrate and internal control. Positivity was determined by the presence of specific IgG fluorescence in successfully transfected cells compared with positive and negative controls. CSF GFAP-IgG was positive at a titer of 1:32, corresponding to a strong positive result according to the laboratory’s CSF CBA-IIFT criteria, whereas serum GFAP-IgG was negative. AQP4-IgG, MOG-IgG, and MBP antibodies were negative in both serum and CSF.

**Figure 1:**
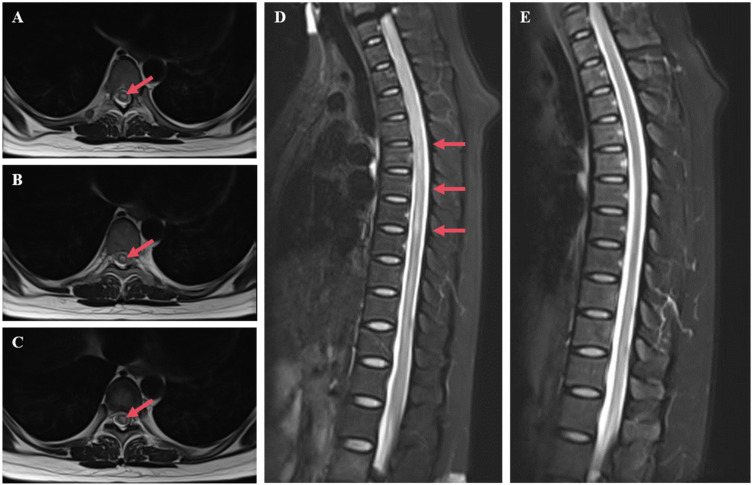
Thoracic spinal magnetic resonance imaging findings. **(A–C)** Initial axial T2-weighted thoracic spinal MRI images at representative lesion levels show centrally located intramedullary hyperintensities, predominantly involving the central/periependymal region of the spinal cord, without obvious cord swelling or mass effect. **(D)** Initial sagittal T2-weighted fat-suppressed thoracic spinal MRI shows discontinuous, patchy, longitudinally extensive intramedullary hyperintensities in the upper-to-middle thoracic cord, approximately from T2 to T6. **(E)** Follow-up sagittal T2-weighted fat-suppressed thoracic spinal MRI obtained 3 months later shows complete resolution of the previous intramedullary lesions. Arrows indicate the spinal cord lesions.

**Figure 2:**
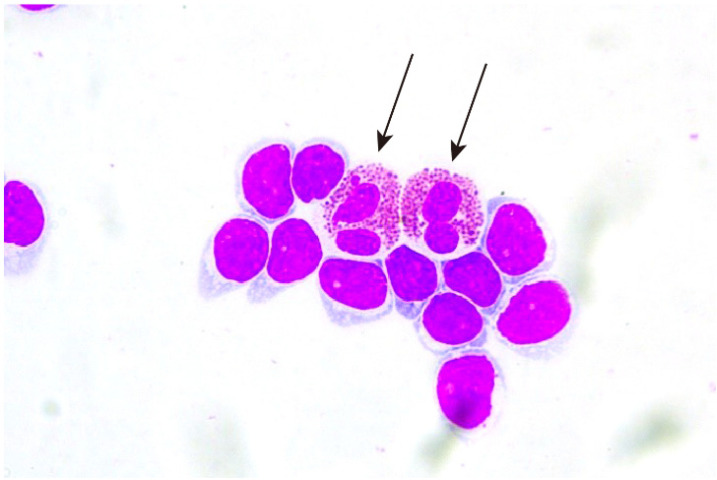
Cerebrospinal fluid cytology showing eosinophils. Eosinophils are indicated by black arrows. May-Grünwald-Giemsa stain, original magnification ×1000.

Taken together, these findings supported a diagnosis of autoimmune GFAP astrocytopathy presenting predominantly as myelitis. The patient received intravenous methylprednisolone 1000 mg daily for 3 consecutive days, followed by tapering by 50 mg every 3 days. When the dose was reduced to 120 mg/day, oral prednisone was initiated at 60 mg/day and then tapered by 5 mg every 2 weeks. After 1 week of treatment, repeat lumbar puncture showed improvement in CSF findings: the opening pressure decreased to 200 mmH2O, the leukocyte count to 145/mm^3^, and the protein level to 867 mg/L. Repeat cytology showed 94% lymphocytes, 5% monocytes, and 1% eosinophils. Dynamic changes in CSF parameters and cytological findings on Day 0 and Day 7 are summarized in **Table 1**. Her fever gradually subsided, and urinary function improved during hospitalization. At the 3-month follow-up, fever and urinary retention had completely resolved. The patient reported normal spontaneous urination and had no recurrent urinary retention. She had no residual neurological symptoms, including limb weakness, sensory disturbance, gait impairment, visual symptoms, cognitive symptoms, or sphincter dysfunction. Neurological examination showed no objective residual deficit, and the modified Rankin Scale score was 0. Follow-up thoracic spinal MRI showed complete resolution of the previous intramedullary lesions (**Figure 1E**). No clinical relapse was observed during corticosteroid tapering and the available follow-up period. Repeat CSF GFAP-IgG testing was not performed. Because the patient achieved complete clinical and radiological recovery and had no recurrence, long-term immunosuppressive therapy was not considered necessary and was not initiated.

**Table 1:** Dynamic changes in cerebrospinal fluid findings before and after corticosteroid treatment.

Time	Appearance of CSF supernatant	Opening pressure (mmH_2_O)	Protein (mg/L)	Glucose (mmol/L)	Chloride (mmol/L)	Microbial cultures	CSF cytology examination				
							WBC (cells/mm^3^)	Lymphocytes (%)	Monocyte (%)	Neutrophil (%)	Eosinophil (%)
Day0	Crystal clear	400	1017	2.31	119	Negative	260	85	5	ND	10
Day7	Crystal clear	200	867	1.88	119	Negative	145	94	5	ND	1

CSF, cerebrospinal fluid; Pro, protein (reference range, 150–450 mg/L); Glu, glucose (reference range, 2.8-4.5 mmol/L); Chloride (reference range, 120–132 mmol/L); WBC, white blood cell (reference range, < 5 cells/mm^3^); ND, not detected.

## Discussion

The present case highlights two atypical features of autoimmune GFAP astrocytopathy: isolated thoracic spinal cord lesions on MRI and eosinophils identified on CSF cytology. Autoimmune GFAP astrocytopathy is a steroid-responsive autoimmune inflammatory disorder of the central nervous system associated with GFAP-IgG, which is most reliably detected in CSF (1, 4). Although both brain and spinal cord abnormalities have been described, the disease is often recognized because of characteristic brain MRI findings, such as linear perivascular radial enhancement, leptomeningeal enhancement, or ependymal involvement (3, 7, 8). In contrast, isolated spinal cord involvement without corresponding brain MRI abnormalities appears less common and may delay recognition, particularly when the presenting syndrome resembles infectious meningitis or meningoencephalitis (9). In the present patient, fever, headache, meningeal irritation signs, markedly inflammatory CSF findings, and a normal brain MRI could easily have redirected the diagnostic workup toward infectious etiologies. However, the presence of CSF GFAP-IgG, the favorable steroid response, and complete radiologic resolution support autoimmune GFAP astrocytopathy as the unifying diagnosis.

The differential diagnosis is particularly important in patients presenting with inflammatory myelopathy. Spinal cord involvement in autoimmune GFAP astrocytopathy is not rare, and reported lesions are often longitudinally extensive, centrally located, and frequently involve the thoracic cord; central canal or leptomeningeal enhancement may also be suggestive (10). These features may overlap with aquaporin-4-IgG-positive neuromyelitis optica spectrum disorder (AQP4-NMOSD), in which longitudinally extensive central cord lesions are also common. However, AQP4-NMOSD more often demonstrates severe longitudinally extensive transverse myelitis with more marked cord swelling and characteristic bright spotty lesions (10–12). By contrast, myelin oligodendrocyte glycoprotein antibody-associated disease (MOGAD) more commonly shows central gray matter involvement with the axial “H-sign” and a predilection for the conus medullaris (10, 13). Clinical context is also informative. Fever, headache, and meningeal irritation signs are more suggestive of autoimmune GFAP astrocytopathy than of AQP4- or MOG-associated myelitis, and marked CSF pleocytosis, elevated protein, and occasionally decreased glucose may further support GFAP astrocytopathy (8, 10). Therefore, when spinal MRI reveals long-segment thoracic lesions without typical NMOSD bright spotty lesions or typical MOGAD features, especially in the setting of inflammatory CSF findings and meningeal symptoms, autoimmune GFAP astrocytopathy should be considered even if brain MRI is unremarkable. Although the patient had no motor weakness, pyramidal signs, or definite sensory level, acute urinary retention together with thoracic intramedullary MRI lesions supported spinal cord involvement affecting bladder autonomic pathways. Therefore, this case is best interpreted as GFAP-IgG-associated myelitis with predominant bladder autonomic dysfunction, rather than classic complete transverse myelitis.

Although CSF eosinophilia and isolated thoracic spinal cord lesions were initially described as two atypical findings, they may be better interpreted as potentially related manifestations of a meningeal–CSF–spinal cord compartment inflammatory process. Lowe et al. reported autoimmune GFAP astrocytopathy with CSF eosinophils in the setting of inflammatory meningoencephalomyelitis (6), whereas Hou et al. described marked CSF eosinophilia in an adult patient with meningitis-like symptoms, normal MRI findings, and CSF GFAP-IgG positivity (5). In the present case, CSF eosinophilia occurred together with meningeal symptoms, markedly elevated CSF opening pressure, urinary retention, and isolated thoracic intramedullary lesions. A concise comparison of these reported CSF-eosinophilic autoimmune GFAP astrocytopathy cases is provided in **Table 2**. Taken together, these observations raise the possibility that CSF-eosinophilic GFAP astrocytopathy may involve the meninges, CSF compartment, and/or spinal cord, rather than representing a purely encephalitic phenotype. However, the number of reported cases remains very small, and this interpretation should be regarded as hypothesis-generating.

**Table 2:** Comparison of reported CSF-eosinophilic autoimmune GFAP astrocytopathy cases.

Feature	Lowe et al.	Hou et al.	Present case
Main clinical pattern	Meningoencephalomyelitis with fever, headache, confusion, and later urinary retention	Meningitis-like presentation with fever, headache, nausea, and neck stiffness	Meningitis-like symptoms with isolated thoracic myelitis and acute urinary retention
Brain MRI	Initially unremarkable on non-contrast MRI; later contrast-enhanced MRI showed basal ganglia perivascular enhancement and diffuse leptomeningeal enhancement	No significant brain MRI abnormality	Unremarkable on conventional and post-gadolinium T1-weighted sequences
Spinal cord involvement	Multifocal cervical and thoracic cord lesions with intramedullary and leptomeningeal enhancement	No definite spinal cord lesion reported	Isolated thoracic intramedullary lesions, approximately T2–T6
CSF eosinophilia	Persistent CSF eosinophils across serial lumbar punctures, approximately 1%–4%	Marked CSF eosinophilia, up to 22%, decreasing after methylprednisolone	10% eosinophils initially, decreasing to 1% after corticosteroid treatment
Treatment response	Improved after corticosteroids; rituximab was added because of severe symptoms and incomplete early recovery	Fever and headache resolved after methylprednisolone; no relapse at 3 months	Clinical, CSF, and radiological improvement after corticosteroids; complete resolution of thoracic cord lesions at 3 months

CSF, cerebrospinal fluid; GFAP, glial fibrillary acidic protein; MRI, magnetic resonance imaging.

A notable aspect of this case is the identification of eosinophils on CSF cytology in association with isolated thoracic spinal cord involvement. In routine clinical practice, CSF eosinophilia more commonly raises suspicion for parasitic infection, fungal infection, neoplastic meningitis, drug-induced meningitis, shunt-related reactions, or other eosinophilic meningoencephalitic disorders. Accordingly, the finding of eosinophils in our patient initially broadened the differential diagnosis toward these more typical etiologies. However, extensive infectious investigations, including CSF culture and metagenomic next-generation sequencing, were negative, and the patient showed clear improvement after corticosteroid therapy. These observations raise the possibility that CSF eosinophilia may occur as part of the inflammatory spectrum of autoimmune GFAP astrocytopathy, although this finding is not disease-specific and requires careful exclusion of alternative causes. Recent case literature suggests that CSF eosinophilia may represent a rare but genuine inflammatory subtype within the spectrum of autoimmune GFAP astrocytopathy (5). Although eosinophils have also been described in AQP4-NMOSD and only rarely in MOG-associated disease, they are not a defining feature of either condition and should not diminish the diagnostic significance of this finding in an appropriate GFAP-IgG-positive context (9, 11, 12).

From a mechanistic perspective, the significance of CSF eosinophils in autoimmune GFAP astrocytopathy remains speculative but intriguing. Because GFAP is an intracellular astrocytic antigen, current evidence favors a predominantly T-cell-mediated process rather than direct antibody-mediated injury (14). From a mechanistic perspective, one possible explanation is that astrocyte-directed inflammation in autoimmune GFAP astrocytopathy disrupts the blood–CSF barrier and promotes cytokine- or chemokine-mediated immune-cell recruitment within the meninges, CSF space, and spinal cord. In this setting, eosinophils may represent a secondary component of the local inflammatory response rather than primary pathogenic effector cells. This interpretation remains speculative and requires validation in larger cohorts with systematic CSF cytology, spinal MRI assessment, and cytokine or chemokine profiling. The recent report of adult autoimmune GFAP astrocytopathy with marked CSF eosinophilia suggested a potential relationship between eosinophil infiltration and disease pathogenesis, and in that case eosinophils declined after corticosteroid treatment in parallel with clinical improvement (5). A similar dynamic was observed in our patient, in whom the eosinophil proportion decreased markedly after immunotherapy together with improvement in CSF parameters and neurologic status. In this single case, the reduction in CSF eosinophils after corticosteroid treatment paralleled improvement in clinical symptoms, CSF inflammation, and spinal MRI abnormalities. However, because eosinophils are highly steroid-sensitive and the observation is based on a single patient, this finding is insufficient to establish CSF eosinophils as a disease activity or treatment-response marker.

Several limitations should be acknowledged. Although the patient had no epidemiological exposure history suggestive of parasitic infection and CSF culture and metagenomic next-generation sequencing were negative, parasite-specific serology and stool examination were not performed. Serum ACE, soluble IL-2 receptor, CSF IgG index, and oligoclonal bands were also not assessed, which limited the completeness of the evaluation for neurosarcoidosis, lymphoproliferative disease, multiple sclerosis, and intrathecal IgG synthesis. Although gynecological ultrasonography showed no evidence of ovarian teratoma or other gynecological mass, systematic whole-body malignancy screening was not performed. In addition, the follow-up period was relatively short, and repeat CSF GFAP-IgG testing was not performed, limiting assessment of long-term relapse risk and serological evolution. Continued clinical and radiological follow-up is warranted because relapse has been reported in autoimmune GFAP astrocytopathy.

In summary, this case illustrates that autoimmune GFAP astrocytopathy may present with isolated thoracic spinal cord lesions on MRI despite prominent meningeal symptoms and unremarkable brain MRI findings. CSF eosinophilia remains non-specific, but in an appropriate clinical context it may serve as an additional clue to GFAP-IgG-associated inflammation after alternative causes have been carefully excluded. Awareness of this combination may help avoid misdiagnosis, prompt earlier GFAP antibody testing, and facilitate timely immunotherapy.

## Data Availability

The datasets presented in this article are not readily available because of ethical and privacy restrictions. Requests to access the datasets should be directed to the corresponding authors.
